# Visual outcome measures in clinical trials of remyelinating drugs

**DOI:** 10.1136/bmjno-2023-000560

**Published:** 2024-02-19

**Authors:** Gioia Riboni-Verri, Benson S Chen, Christopher E McMurran, Gregory J Halliwell, J William L Brown, Alasdair J Coles, Nick G Cunniffe

**Affiliations:** 1 Department of Clinical Neurosciences, University of Cambridge, Cambridge, UK; 2 Cambridge Clinical Vision Laboratory, University of Cambridge, Cambridge, UK; 3 Department of Medical Epidemiology and Biostatistics, Karolinska Institutet, Stockholm, Sweden; 4 Clinical Outcomes Research Unit (CORe), University of Melbourne, Melborune, Melborune, Australia

**Keywords:** MULTIPLE SCLEROSIS, CLINICAL NEUROLOGY, VISUAL EVOKED POTENTIALS, NEUROOPHTHALMOLOGY, MYELIN

## Abstract

One of the most promising approaches to delay, prevent or reverse disability progression in multiple sclerosis (MS) is to enhance endogenous remyelination and limit axonal degeneration. In clinical trials of remyelinating drugs, there is a need for reliable, sensitive and clinically relevant outcome measures. The visual pathway, which is frequently affected by MS, provides a unique model system to evaluate remyelination of acute and chronic MS lesions in vivo and non-invasively. In this review, we discuss the different measures that have been used and scrutinise visual outcome measure selection in current and future remyelination trials.

## Introduction

Multiple sclerosis (MS) is a chronic immune-mediated disorder of the central nervous system (CNS) characterised pathologically by inflammation, demyelination and axonal loss.^1 2^ Approximately 85% of people with MS present with relapsing-remitting MS (RRMS), in which episodes of acute focal demyelination are followed by variable degrees of recovery.^3^ Left untreated, around 80% of these patients will accrue irreversible disability (secondary progressive MS, SPMS), having developed chronic demyelination and neurodegeneration.^4^ Meanwhile, 15% of people living with MS have progressive disability—typically without discrete relapses—from the outset, in what is described as primary progressive MS.^5^

Current licensed MS disease-modifying treatments act by modulating the inflammatory component of the illness and are deployed in RRMS to reduce the frequency of relapses^2^ and the rate of conversion to SPMS.^6^ However, only siponimod and ocrelizumab have shown any effect on reducing disability accrual in progressive forms of the illness,^7 8^ while their benefit appears modest and restricted to those with ongoing inflammatory activity. As such, the greatest unmet clinical need for people living with MS is treatments that prevent the axonal and neuronal damage responsible for permanent disability.^9^

As remyelination restores nerve conduction and limits axonal degeneration in MS,^10^ therapies capable of enhancing endogenous remyelination are rapidly emerging as a leading strategy to delay, prevent or reverse disability progression.^2^ Fundamental to this has been an improved understanding of the biology of remyelination, which primarily relies on the activation, migration, proliferation and differentiation of oligodendrocyte progenitor cells (OPCs) into new myelinating oligodendrocytes.^11^ In people with MS, endogenous remyelination via OPCs fails, and the rate-limiting step appears to be an inability of OPCs to differentiate.^12 13^ While evidence also points to a role for established oligodendrocytes^14 15^—and a small contribution from subventricular zone progenitors^16^—in the repair process,^17^ therapies with the potential to enhance OPC differentiation are the leading candidates at present, and several are being deployed in phase 2 trials.^18–23^

However, outcome measure selection poses a major translational challenge to these early-phase clinical trials.^2^ In preclinical studies, high-resolution transmission electron microscopy of histological sections of remyelinated tracts has been established as the gold standard since the 1970s.^24^ Unfortunately, a similarly robust measure in clinical studies is lacking. An ideal outcome would be sensitive and specific to the biological effects and pathology of remyelination, be simple and inexpensive to measure in a rigorous manner across multiple sites, be measurable in all people with MS and be strongly associated with patient experience and clinical efficacy. No current test meets that charge, and studies are increasingly relying on a combination of neurophysiological and/or imaging-based assessments.^18 19 21 23 25^ Although some neurophysiological and imaging-based assessments have been identified to be specific to biological changes in myelin, there is a challenge in translating such promise at the clinical level. The current model is that these measures can be deployed to demonstrate biological remyelination in short-duration early-stage clinical trials, before giving way to less sensitive, but potentially more clinically meaningful, measures of disability change in long-duration phase 3 trials.

The myelin-sensitive MRI sequences include myelin water fraction (MWF), diffusion tensor imaging (DTI) and magnetisation transfer ratio (MTR)—with changes in MTR showing the most promise for detecting lesion-level remyelination.^18 23 26^ However, these MRI-based techniques vary in their pathological specificity. Positron emission tomography (PET) imaging of myelin and oligodendrocytes has been used to quantify myelin,^27^ but the availability of this technique, radiation and the lack of established and specific radioligands are significant barriers. A blood biomarker of remyelination is an unmet need; established fluid biomarkers such as neurofilament light chain (NfL) and glial fibrillar acidic protein (GFAP) are reflective of axonal health but would be only indirectly impacted by remyelination.^28 29^

Visual outcome measures have therefore become increasingly important in remyelination trials.^18 19 21^ There are several reasons for this. First, the visual pathway is frequently involved in the course of MS: 20% of people with MS present with acute optic neuritis (AON) as their first symptom,^30^ approximately half of people with RRMS have evidence of previous optic neuritis (ON),^31^ while optic radiation lesions are seen in nearly 70%.^32^ Second, the recovery from inflammatory demyelination of the visual pathway is seldom complete.^33^ And third, visual evoked potentials (VEPs), visual fields, visual acuity and optical coherence tomography (OCT) are reliable and inexpensive measures that can be readily used in a clinical trial, with VEPs emerging as the most sensitive and responsive to remyelination.

Using these measures as primary endpoints does introduce a bias to remyelination exclusively in the visual pathway. And there are significant pathological differences between acute and chronic MS lesions which need to be scrutinised in the planning of a trial: in the acute inflammatory stages, infiltration with activated macrophages, microglia, lymphocytes and reactive astrocytes is seen, whereas chronic lesions are typified by subsidence of inflammatory pathology with oligodendrocyte and axonal loss and surrounding astrocytic scar formation.^34^ Acute and chronic MS lesions in the visual pathway may vary in their remyelinating capacity, and so the ages of lesions need to be considered in trial design. Nevertheless, evaluations of lesions in the visual pathway remain the leading way to test functional remyelination in people living with MS.^30 35^

In this review, we discuss the different measures of visual structure and function that have been used in remyelination clinical trials and scrutinise future directions for clinical trials of remyelination in people with MS.

## Visual evoked potentials

### Full-field pattern-reversal VEP

**Figure 1:**
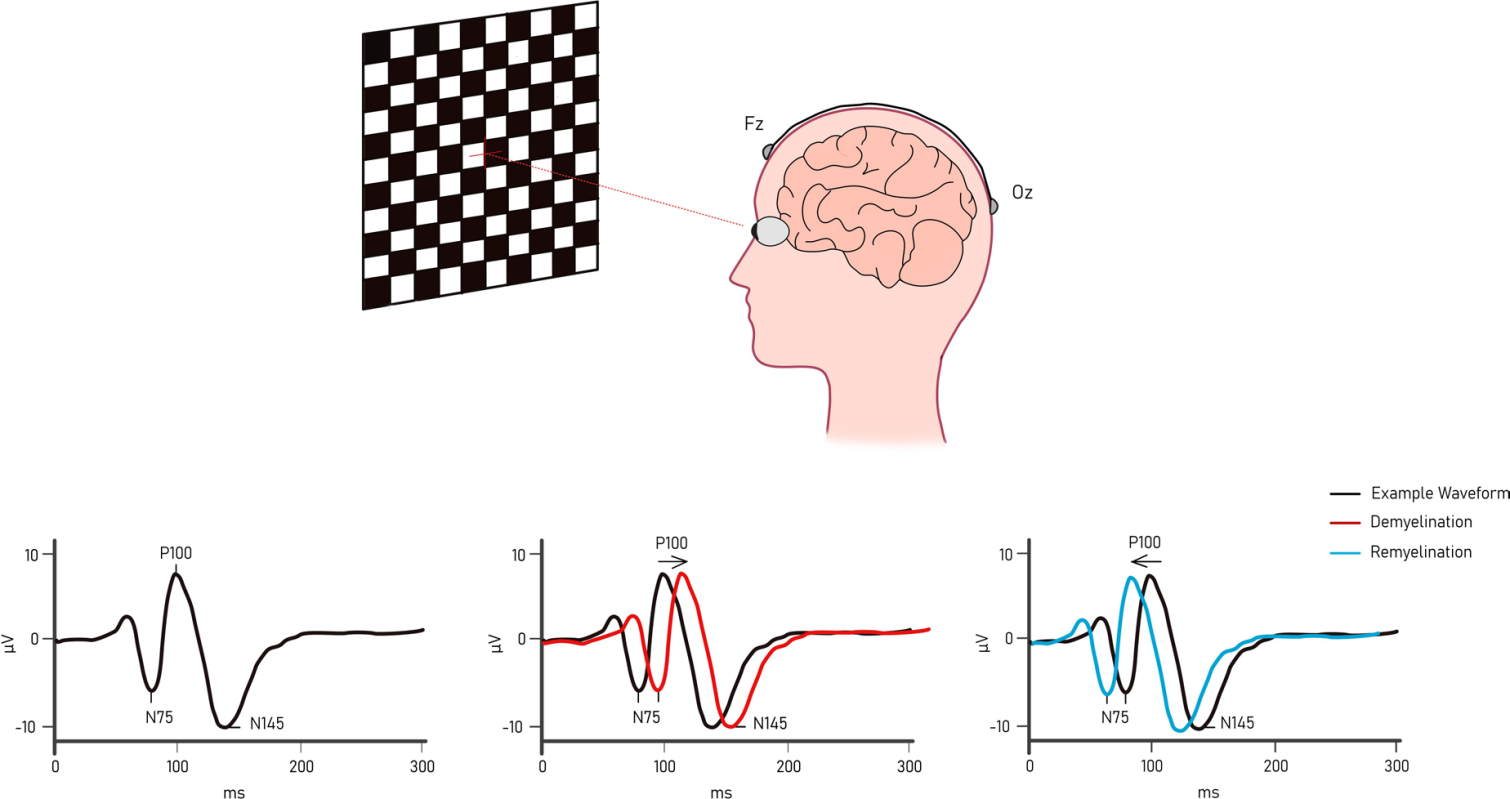
Visual evoked potential waveform elicited by a (usually 2 Hz) pattern reversing stimulus. The negative pole electrode (Fz) is placed on the upper forehead and the positive pole electrode (Oz) in the midline of the occipital scalp (International Society for Clinical Electrophysiology of Vision standards). The resulting signal comprises a negative component at a peak time of about 75 ms (N75), a larger positive component at 100 ms (P100) and a more variable negative component at about 140 ms (N145). Figure created by the authors. Use permitted in this publication.

VEPs are generated by the primary (striate) visual cortex and represent the combined activity of postsynaptic and cortical potentials in response to a visual stimulus.^36^ While a variety of different stimulus paradigms are possible, the optimum practice in MS is to use a repetitive reversing checkerboard-patterned stimulus (the full-field pattern-reversal VEP, ff-VEP).^37^ This is recorded by channels formed between occipital and frontal electrodes^38^ and distinguished from the electrical background by a process of signal amplification and averaging to generate a single waveform (figure 1).^39^ The amplitude of the resultant signal has been hypothesised to reflect the number of functional fibres along the stimulated visual tract, serving as a parameter of axonal loss.^40 41^ Meanwhile, the latency of the generated waveform is consequent on the speed of conduction of the fastest conducting fibres in the retino-geniculate-striate pathway.^42^ It has long been established that an increase in latency follows demyelination. But, most pertinently, convergent histological data across different models of experimental demyelination have shown that the subsequent reduction in latency following demyelination directly indicates remyelination, rather than ion channel redistribution, resolution of conduction block or plasticity.^42 43^ Therefore, changes in VEP latency have been deployed as an in vivo biomarker of myelin repair and frequently used as a primary endpoint in clinical trials of putative remyelinating agents.

However, for the VEP to be used in remyelination trials, it must rely on a pathologically appropriate target population of people with delayed VEP latencies. Furthermore, changes in VEP latency must be placed in the context of the potential confound of spontaneous VEP recovery following AON; following AON, VEP latencies are prolonged (>125 ms) and follow a period of spontaneous recovery that is most pronounced in the first 3–6 months but can last up to 2–3 years.^44^ Trialists therefore must choose between AON—when the approximate time of onset of demyelination is known—and chronic stable optic neuropathy, when there have been no recent instances of clinical AON.

#### The use of full-field VEP to measure remyelination of chronic lesions

The ReBUILD (NCT02040298) study was a single-centre, double-blind, randomised, placebo-controlled, phase 2, crossover trial, which investigated the effect of clemastine—a first-generation antihistamine capable of stimulating OPCs to differentiate into remyelinating oligodendrocytes^45 46^—on the ff-VEP of 50 people with RRMS and chronic demyelinating optic neuropathy.^21^ This study was carefully designed with the limitations of the ff-VEP in mind. Participants both with and without a history of clinical ON were eligible, but each had to have a VEP P100 latency ≥118 ms VEP in at least one eye, while ON events were restricted to have occurred no less than 5 years prior to randomisation in an affected eye. An additional criterion was that included eyes would have a retinal nerve fibre layer (RNFL) thickness >70 µm, in the expectation this indicated sufficient scaffolding of existing denuded axons to allow for remyelination.^21^ In a double-blind crossover design, 25 participants were given 5.36 mg of clemastine twice daily for 90 days followed by placebo for 60 days (group 1), while a further 25 participants were given placebo for 90 days followed by clemastine for 60 days (group 2). The trial reported a statistically significant reduction of 1.7 ms/eye (95% CI 0.5 to 2.9, p=0.0048) in P100 latency in the crossover model. Yet, as crossover trials are susceptible to a carryover effect driven by a sustained treatment effect of active compound following crossover to control epochs, it was concluded that this effect was underestimated: a 3.2 ms reduction in P100 latency was reported. An effect was observed in both participants with and without history of ON, though a post hoc analysis suggested that participants with previous clinical episodes had a more pronounced response.^21^ However, it should be stated that an improvement of 3.2 ms in P100 latency is unlikely to be reflected in a patient’s visual acuity or quality of life. Rather, this change suggests that remyelination did occur, though any clinical benefit to the participant is likely to emerge in the long term. The ReBUILD trial design did have limitations. Foremost, 75 patients were excluded because their screening VEPs did not meet the threshold latency of 118 ms. Additionally, while the crossover trial design had advantages from the perspective of recruitment (as all participating patients received the active drug during an epoch), the same design requires the active drug and its effects to be rapidly washed out between epochs—a requirement that is not sensible if the aim of the trial is to detect long-term structural remyelinating changes. It remains to be seen whether this result can be reproduced in a larger, more heterogeneous population.

In support of analyses of participants with delayed P100 latency at baseline, without a recent history of acute ON, the CCMR One trial similarly reported a statistically significant improvement in ff-VEP P100 latency among eyes with baseline values >118 ms.^18^ This trial assessed the effect of bexarotene —an agonist of the retinoic acid receptor and a known positive regulator of OPC differentiation^47^—in people with RRMS.^18^ A delay in baseline VEP latency was not a selection criterion for CCMR One, but a prespecified exploratory analysis identified 43 eyes (50% of total eyes) with a delayed (>118 ms) baseline VEP latency and without a history of AON in the past 5 years. Within this population, a significant reduction in P100 latency between bexarotene and placebo was observed (−4.75 ms (95% CI −8.80 to –0.71, p=0.032)). Although bexarotene was poorly tolerated due to side effects at the 300 mg/m^2^ dose, this study further highlighted the potential of ff-VEP as a remyelination outcome measure.

#### The use of full-field VEP to measure remyelination of acute lesions

An alternative approach in remyelination trial design is to study treatment effects on the ff-VEP in participants following an episode of AON. There is a compelling rationale for this: remyelination may be more successful in acute lesions, given the abundance of intact axons alongside a pro-reparative microenvironment with various positive regulators of OPC differentiation.^13 48–51^ Acute lesions may therefore represent an optimal window of opportunity for the use of a remyelination-promoting drug.^52^ Given that the clinical time of onset of AON is known, this approach potentially also overcomes the confound of varying lesion ages in the trials described above.^30^

The RENEW (NCT01721161) study of opicinumab—a monoclonal antibody against LINGO-1, a negative regulator of OPC differentiation—recruited 82 participants with a first episode of AON.^19^ This was a randomised, double-blind, placebo-controlled, phase 2 study which tested the effect of opicinumab on ff-VEP P100 latency recovery in the affected eye, referenced to the unaffected eye, over 24 weeks of treatment. The mean treatment difference between opicinumab and placebo was −3.5 ms (95% CI −10.6 to 3.7, p=0.33) in the intention-to-treat population (though −7·6 ms in the per-protocol population (95% CI −15.1 to 0.0, p=0.050)). In a similar vein, clemastine is now being tested in the ReCOVER trial (NCT02521311) which will test its effect among 90 participants diagnosed with AON.

### Multifocal VEP

There are, however, limitations to the ff-VEP experimental technique. ff-VEPs generate a waveform representing stimulation of the entire visual field, and so represent the summation of all produced postsynaptic potential dipoles. The retinotopic projection to the visual cortex means that the upper visual field projects to the lower bank (lingual gyrus) of the sulcus calcarinus, while the lower visual field projects to the upper bank (cuneus gyrus). As these face each other, the cortical dipoles from the upper and lower hemifields are almost opposite, resulting in a cancellation effect of amplitude in the unaffected eye.^30 53^ Accordingly, another consequence of damage to a discrete location in the visual pathway, such as a MS lesion, can be that the recorded signal appears larger due to less cancellation effect.^54^ Further limitations of ff-VEP are that macular over-representation in the visual cortex weights any latency abnormalities significantly to those in the central field.^55 56^ This problem is compounded by the conventional electrode placement (frontal-occipital) which favours the response from the lower visual field.^57^

**Figure 2:**
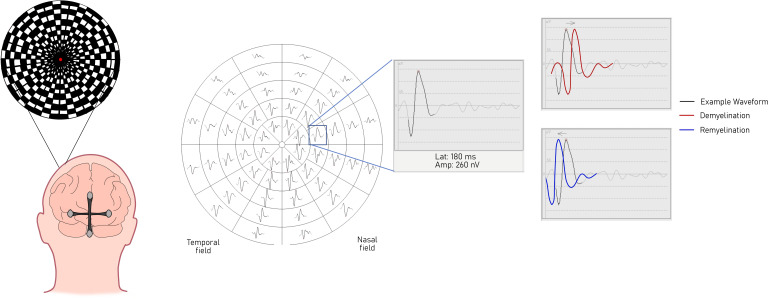
In a multifocal visual evoked potential, 56 cortically scaled segments of flash pattern stimuli simultaneously reverse in pseudorandom sequence to elicit a unique signal from each. Electrode array placement comprises four electrodes—two lateral electrodes placed at O1 and O2, a third electrode placed above the inion in the midline and a fourth below the inion in the midline. Figure created by the authors. Use permitted in this publication.

Multifocal VEP (mf-VEP) mitigates against these problems by stimulating up to 60 individual regions of the visual field simultaneously and extracting the unique signals corresponding to each^39 54^ (figure 2). This allows for a potentially more precise analysis of latency and amplitude abnormalities in people with optic neuropathy.^58 59^ There is a clear relationship between VEP latency and lesion length in the optic nerve^60^ and lesion volume in the posterior visual pathway.^61^ mf-VEP measures are also highly stable and reproducible. In a recent cohort study of 50 RRMS patients with repeated measures over 12 months,^33^ there was no significant latency change.

The best example of mf-VEP in a remyelination study, to date, was an exploratory substudy of the RENEW trial led by Klistorner and colleagues.^25 62^ Their per-protocol analyses showed trends to latency improvement (mean change of −11.78 ms between opicinumab and placebo (95% CI −24.28 to 0.73, p=0.06)) and amplitude recovery (mean gain vs placebo was 22.32 nV (95% CI −1.26 to 45.89, p=0.06)), but significant variation between subjects led the study authors to conclude that they were underpowered with only 39 participants. An additional substudy (RENEWED)^63^ demonstrated that trends in latency improvement in the opicinumab group were sustained 2 years following the end of trial participation (mean change of −15.1 ms between opicinumab and placebo (95% CI –33.4 to –5.8, p=0.01)). There was also observed to be a positive association between baseline latency and degree of latency recovery in the opicinumab group; the remyelinating effect of opicinumab treatment was seemingly proportional to the initial degree of demyelination. Although the sample size was small (n=18), this result is reassuring that remyelinating therapies can induce long-term structural changes.

Although mf-VEP requires more time to be performed and can therefore be demanding for people with MS, these results have supported mf-VEP being deployed in phase 2 trials. For instance, the CCMR Two (NCT05131828) phase 2 placebo-controlled trial of the combination of metformin and clemastine uses the change in mf-VEP latency and amplitude as a secondary outcome measure.

## Optical coherence tomography

**Figure 3:**
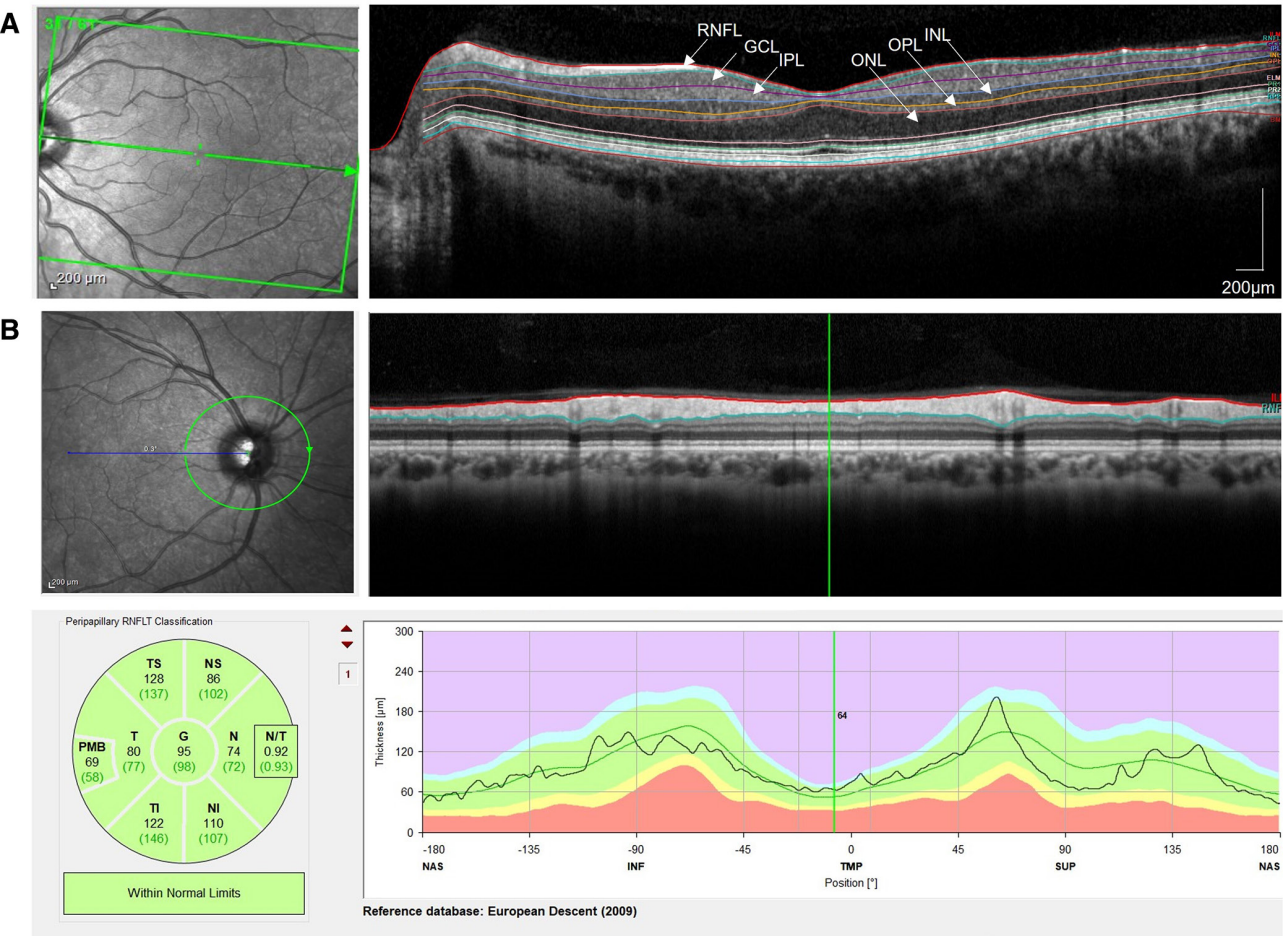
(A) A macular optical coherence tomography (OCT) section illustrates the retinal layers comprising the retinal nerve fibre layer (RNFL), ganglion cell layer, inner plexiform layer, inner nuclear layer, outer plexiform layer and outer nuclear layer. (B) A ring B scan of 3 mm around the optic disk (art 100) illustrating peripapillary RNFL. Both using Heidelberg Spectralis (Heidelberg engineering, Heidelberg, Germany) OCT. RNFL, retinal nerve fibre layer; GCL, ganglion cell layer; IPL, inner plexiform layer; INL, inner nuclear layer; OPL, outer plexiform layer; ONL, outer nuclear layer; RNFLT, retinal nerve fibre layer thickness; PMB, papillomacular bundle; TS, temporal superior; NS, nasal superior; T, temporal; G, global; N, nasal; TI, temporal inferior; NI, nasal inferior; NAS, nasal; INF, inferior; TMP, temporal, SUP, superior.

OCT is a non-invasive technique that allows cross-sectional imaging and segmentation of retinal structures.^64^ The retinal ganglion cells (RGCs), whose cell bodies are found in the ganglion cell layer (GCL) and axons found in the RNFL,^65^ provide a particularly valid window for quantifying axonal and neuronal loss in people living with MS (figure 3). Depending on the OCT platform, the GCL is sometimes combined with the inner plexiform layer (IPL)—which represents the dendrites of RGC—to assess GCIPL. The long axons of the RGC coalesce at the optic nerve and then travel posteriorly through the optic chiasm, optic tract and finally the lateral geniculate body, where they synapse with neurons that project to the visual cortex.

If severe enough to cause axonal damage, any disease process involving the visual pathway can potentially lead to loss of RGC which manifests with thinning of RNFL and GCL on OCT.^66^ The thickness of both layers can be measured in the macula or around the optic nerve in the peripapillary region. It is consistently reported that macular GCL (mGCL) and peripapillary RNFL (pRNFL) thicknesses are significantly reduced in the eyes of people with MS, even without a history of ON.^67 68^ In some patients with retrochiasmal MS lesions, retrograde trans-synaptic neurodegeneration can give rise to homonymous hemimacular atrophy of the mGCL.^69^ There is increasing observational evidence that loss of macular GCIPL (mGCIPL) thickness in people living with MS correlates with MS lesion activity in the visual pathways.^70–72^ Indeed, loss of pRNFL thickness has also been correlated with brain imaging evidence of atrophy in people with MS, supporting a pathobiological link with neurodegeneration.^73^ As a result, OCT measures have been advocated as a potential outcome measure in neuroprotective treatment trials.^74 75^

The selection of an OCT outcome in remyelination trials requires insight into the time course of recovery in the event of AON. Typically, pRNFL measures are in fact elevated in the acute stages due to axoplasmic flow stasis.^72^ With time, there is progressive RNFL loss, with the majority of thinning occurring between 3 and 6 months after ON.^76–78^ In total, a reduction of ~20 µm in pRNFL follows AON in people with MS; the more severe the attack of ON, the greater the loss of pRNFL and mGCIPL.^72 79^ The decline is not linear, with rapid thinning occurring over the first 6 weeks before a more gradual decline over around 200 days.^37^ Meanwhile, measures of mGCIPL are generally unaffected by acute inflammation and oedema in ON, but thinning begins as soon as 4 weeks after symptom onset and is complete by 3 months.^80^ Therefore, changes in mGCIPL is a preferred measure for quantifying early degeneration following ON, while changes in pRNFL thickness are perhaps best reserved for when at least 6 months remote from ON.

Consequently, in RENEW, which centred on the treatment response to opicinumab in AON, change in GCIPL thickness was a secondary efficacy endpoint.^19^ There was no statistically significant difference in mean change in GCIPL thickness (at week 24 vs fellow eye at baseline) between study participants treated with opicinumab and those treated with placebo. However, it was observed that most GCIPL thinning occurred prior to the first administration of opicinumab, and no further thinning was observed following the second dose of opicinumab at week 4. Thus, it could be argued that opicinumab halted axonal degeneration as a result of a neuroprotective effect of remyelination or that opicinumab had no direct effect on neuroprotection.

For OCT outcomes to be used in trials, and to power a study based on halting pRNFL or mGCIPL degeneration, it is important to understand how atrophy progresses in the retina in people with MS. Studies have shown some variability in longitudinal changes in OCT measures, for instance, with annual pRNFL atrophy rates ranging from −0.36 µm to −1.49 µm per year.^68 81^ While these rates of retinal atrophy are greater than healthy controls,^82^ they need to be interpreted in light of the variability of the OCT technique, which can be associated with changes up to 5–6 µm in RNFL.^72^ Given such variability, sample sizes must be large to detect a treatment effect over a clinical trial lasting less than a couple of years. For example, in the MS SMART clinical trial cohort of people with SPMS, annualised atrophy rates were observed for each of pRNFL (−0.52 µm) and GCIPL (−0.42 µm), but their sample size estimations indicated in the region of 300 participants would be required per arm to detect a 50% effect size with 90% power.^83^ In addition, changes in the pRNFL and mGCIPL are more pronounced early in the disease course,^84^ suggesting that neuroaxonal loss occurs early in MS or that there is a ‘floor effect’ due to challenges in detecting new pRNFL or mGCIPL thinning on a background of pre-existing neuroaxonal damage. Indeed, other non-neurological disease processes such as glaucoma can also give rise to loss of pRNFL or mGCIPL, which further complicates interpretation.

Perhaps a more eloquent use of OCT in remyelination trials is to select eyes with sufficient preservation of axons to maximise the chance of detecting remyelination. In ReBUILD, Green and colleagues used pRNFL thickness at baseline to identify those eyes with significant axonal loss, excluding those eyes with <70 µm.^21^ While there is large intersubject variability in OCT parameters, this point is viewed as a threshold, below which marked reductions in visual function are seen.^76^ The selection of eyes with sufficiently preserved VEP amplitude (ie, axonal health) could be an alternative approach to maximising the detection of remyelination. However, the use of VEP amplitude as a selection criterion would require consensus over what is a clinically relevant minimum threshold of amplitude. Overall, although the availability and reproducibility of OCT are increasing,^85^ in the context of remyelination trials, OCT remains secondary to visual electrophysiology and MRI.

## Visual acuity

Tests of visual acuity and colour vision are an accessible, validated and functionally relevant measure of the resolving capacity of the visual system. However, similar to OCT, changes in visual acuity and colour do not specifically reflect myelin repair. Instead, changes are more likely responsive to the axonal protection incurred by remyelination, though it is conceivable that the resolution of conduction block in the parvocellular pathway could also impact acuity.

Acuity is assessed in a standardised fashion using Snellen or Early Treatment Diabetic Retinopathy Study (ETDRS) charts, in which a series of optotypes are arranged in lines of decreasing size. The ETDRS chart is the most widely deployed in clinical trials as it has several advantages over Snellen charts. It consists of 14 rows of 5 letters, which are consistently spaced in proportion to letter size. Each row has 5 ‘Sloan’ letters, and each line is sized in equal logarithmic intervals (0.1) of the minimum angle of resolution; thus, with each letter correctly identified, there is a reduction in the logMAR of 0.02.

However, a normal high-contrast visual acuity (HCVA) assessment may miss MS-related visual pathology; in the ON treatment trial (ONTT), low-contrast visual assessments were consistently more sensitive than HCVA.^86^ While the ONTT used Pelli-Robson charts, which test letters of constant size but decreasing contrast,^87^ the favoured measurement for MS research is now Sloan low-contrast letter acuity (LCLA) in which letters of varying intensity of grey are read against a white background (for instance, 2.5% contrast).^88 89^ LCLA testing with Sloan letter charts has been incorporated into several remyelinating trials to date. In both ReBUILD and RENEW, LCLA was deployed as a secondary outcome measure. In ReBUILD, monocular testing of visual acuity with 2.5% contrast revealed a 0.9 letter per eye improvement (95%CI −0.1 to 1.9, p=0.085) in the crossover analysis and a 1.6 letter per eye improvement in the delayed treatment model (95%CI 0.2 to 3.0, p=0.022) when on treatment.^21^ Meanwhile in RENEW, monocular assessment via 1.25% and 2.5% Sloan letter charts showed no significant change between placebo and opicinumab groups.^19^ Although preferred, there are still several limitations to LCLA highlighting why this measure is not usually used unaccompanied as an outcome measure in trials. Technical factors such as optimal refraction, backlighting and luminance of the testing environment can affect LCLA results. Similarly, the 2.5% and 1.25% Sloan charts may be subject to ‘floor’ and ‘ceiling’ effects such that the 1.25% contrast may preclude from scoring any letters at baseline and 2.5% contrast may be too permissive to people with MS such that no true improvement in score will be evaluable over time.^35 88^ It is also possible that some will show a learning effect; for instance, in the ReBUILD study, LCLA assessments were recognised to be confounded by a learning effect that was observed in patients over the course of the trial.^21^

## Colour vision

Patients with MS have profound abnormalities in colour discrimination, which strongly correlate with RNFL thickness and traditional measures of acuity^90^ ; colour vision has been advocated as a candidate biomarker of disease progression. A variety of colour vision assessments are available to the clinician: Ishihara pseudoisochromatic (Ishihara) and Hardy Rand Rittler plates, other colour arrangement tests such as the Farnsworth Dichotomous test (Panel D-15), the Lanthony D-15 desaturated test (D-15d), colour matching tests (eg, anomaloscopes) and the Farnsworth-Munsell (FM) 100 hue test, among others. Yet, colour vision testing in interventional studies of ON and MS has traditionally relied on the use of Ishihara pseudoisochromatic plates (Ishihara) or the FM 100 hue test.^91–93^ Both have limitations. The Ishihara test allows the assessment of colour deficiencies exclusively along the red-green axis without detecting defects along the blue-yellow axis, whereas the FM 100 hue test is impractical in a clinical setting and time-consuming. Computer-based tests such as the Cambridge Colour Test, which evaluates chromatic threshold along the protan, deutan and tritan confusion axes, have therefore emerged as an alternative quantitative and qualitative test of colour vision performance.^94^ To date, there are a handful of trials that have assessed colour vision in participants with ON as an outcome measure.

In a randomised controlled study assessing simvastatin in patients with clinically diagnosed ON,^92^ 64 patients were assessed on colour vision using the Lanthony desaturated 15-hue test and Velhagen pseudoisochromatic plates. No treatment effect between placebo and simvastatin groups was detected, although colour perception was slightly better in the simvastatin group. A more recent study of phenytoin—a voltage-gated sodium channel inhibitor—in patients with acute ON assessed colour vision by FM 100 hue test as a secondary clinical endpoint.^91^ No significant treatment effect was noted. To our knowledge, no previous remyelination trial has used colour vision as an endpoint, and there is uncertainty in which test would be most sensitive to remyelination.

It is our practice that LCLA and colour vision be deployed as tests of functional visual integrity, but we feel it should be used in combination with other tests of visual structure and function within a remyelination trial.

## Oculography

An alternative strategy for effectively measuring remyelination and neuroprotection in people with MS is to leverage opportunities provided by assessments of the efferent visual system. Eye movement abnormalities are common in people with MS and can be rapidly, precisely and non-invasively assessed using high-frequency infrared oculography.

**Figure 4:**
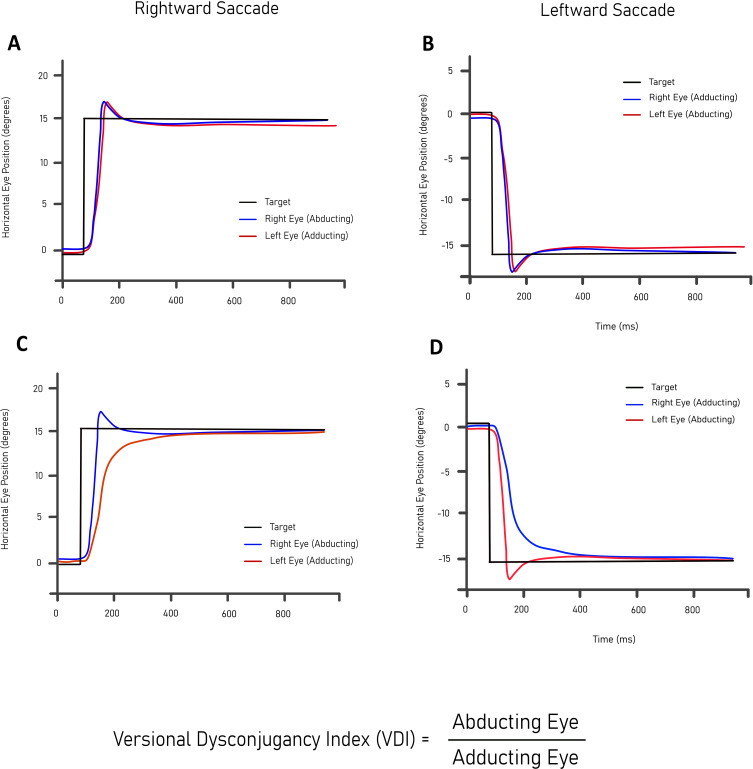
(A,B) An example of a healthy volunteer leftward and rightward saccade. (C,D) An example of internuclear ophthalmoplegia in a leftward and rightward saccade.

Internuclear ophthalmoplegia (INO) is the archetypal eye movement disorder in people with MS and is present in 24%–55% of patients.^95^ This results from damage to the densely myelinated medial longitudinal fasciculus (MLF), which links the contralateral abducens and ipsilateral oculomotor nuclei to ensure synchronous abduction and adduction of the eyes.^96^ An INO comprises failure of adduction of the eye ipsilateral to the MLF lesion, which varies in degree and can be quantified by measuring interocular dysconjugacy.^97^ An improvement in the degree of INO, if present, would be a strong marker of remyelination. Evidence to support this association comes from a randomised, double-blind, placebo-controlled trial of fampridine by Kanhai and colleagues.^97^ Fampridine is not known to promote remyelination but is understood to enhance nerve conduction velocity by blocking voltage-gated potassium channels. As its primary outcome measure, this study used versional dysconjugacy indices for both peak velocity (PV-VDI) and first-pass amplitude (FPA-VDI), calculated in each case by dividing the value of the abducting eye by that of the adducting eye (figure 4). It was found that fampridine improved saccadic eye movements, and the effect on both PV-VDI and FPA-VDI was statistically significant compared with placebo. This study provided evidence that, like ON, chronic INO represents an MS-relevant pathology that can be assessed in trials. Indeed, in a current phase 2 trial of clemastine, this represents the primary outcome measure (NCT05338450), with fampridine response being used as a measure of axonal integrity in the MLF, not dissimilar to the RNFL cut-offs in other trials. However, also akin to trials using VEPs, in the original study, 42 out of 66 people screened did not have a detectable INO, and therefore investigators have to contend with a high screening failure rate.

Other reported eye movement abnormalities in MS include fixation instability,^98^ prolonged saccadic latency,^99^ reduced saccadic velocity^100^ and higher error rates on the antisaccade task.^101^ The recruitment of brain regions in saccade generation is highly task specific, and given that these networks are often myelinated, one might hypothesise that eye movement assessments are a promising additional outcome in remyelination trials.^102^ Standardised protocols using infrared oculography, such as the Demonstrate Eye Movement Networks with Saccades (DEMoNS) protocol,^103^ now exist enabling multicentre measurement and analysis. One particularly promising technique is the measurement of double-step saccades—people with MS make significantly fewer correct double-step saccades than healthy controls, and these errors strongly correlate with grey matter atrophy—which have a strong case to be included in remyelination trials.

## Patient-reported outcomes

Patient-reported outcomes and visual function questionnaires such as the 25-item version of the National Eye Institute Visual Function Questionnaire might be additionally useful in grounding changes in visual outcomes in what is clinically meaningful to the patient. While not specific to remyelination, visual quality of life correlates with LCLA and structural measures at OCT^104 105^ and so should be recommended for trials using visual outcome measures.

## Conclusions and future directions

A particular challenge in translating promising preclinical research into remyelination trials is uncertainty in the optimum outcome measures to employ (table 1).^22 106^ Visual outcomes have consistently shown their value and carry advantages over myelin-sensitive MRI sequences. They are sensitive to changes in nerve structure (ie, OCT) and nerve function (ie, VEP). They can be rapidly, non-invasively and inexpensively assessed across multiple sites and may allow for recruitment of smaller sample sizes compared with patient-level analyses using myelin-sensitive MRI sequences.

**Table 1:** : Summary of terminated or active clinical trials of putative remyelination promoting drugs that use ff-VEP and mf-VEP, OCT, LCLA, colour vision or eye movements as primary, secondary or exploratory outcome measures

Trial name	Trial description	Primary outcome measure	Secondary and exploratory outcome measures	Status/result
ReBUILD (NCT02040298)	Phase 2, randomised, placebo-controlled, double-blind crossover trial of *clemastine fumarate* in 50 people with relapsing-remitting multiple sclerosis (RRMS) and chronic stable optic neuropathy	Change in ff-VEP P100 latency	Change in visual acuity using high-contrast letter acuity (HCLA) and 2.5% Sloan low-contrast letter acuity (LCLA); change in RNFL thickness	Completed. Statistically significant reduction in latency of 1.7 ms in crossover model and 3.2 ms in delayed treatment analysis^21^
RENEW (NCT01721161)	Phase 2, randomised, placebo-controlled, double-blind trial of opicinumab in 82 people with acute ON	Change in ff-VEP P100 latency in affected eye at week 24 versus unaffected eye at baseline	Change in RGCL/IPL thickness in affected eye at week 24 versus unaffected eye at baseline; change in Sloan 2.5% and 1.25% LCLA in affected eye at week 24 versus unaffected eye at baseline	Completed. Statistically significant latency improvement of −7.6 ms in per-protocol analysis^19^
MS-ON (NCT02220244)	Phase 2, randomised, placebo-controlled, double-blind trial of biotin/MD10003 for treatment of chronic visual loss related to ON in 93 people with progressive MS	Change in visual acuity at 100% contrast from baseline to month 6	Change in ff-VEP P100 latency; change in RNFL thickness	Completed. No significant changes^115^
CCMR One	Phase 2, randomised, placebo-controlled, double-blind trial assessing the safety and tolerability of bexarotene in 52 people with RRMS	Adverse events and withdrawals attributable to bexarotene; change in mean lesional MTR for those lesions with a baseline MTR below the within-participant median	ff-VEP P100 latency change	Completed. Statistically significant latency improvement of −4.06 analysis of those eyes with delayed (>118 ms) baseline P100 latency^18^
NCT01451593	Phase 2, randomised, placebo-controlled, double-blind trial of neuroprotection with phenytoin in acute ON	Change in RNFL thickness in affected eye at month 6 versus unaffected eye at baseline	Change in Sloan 2.5% and 1.25% charts; change in colour vision (FM 100 hue test); change in ffVEP latency and amplitude; change in MRI T2, MTR, DTI sequences	Completed. The adjusted mean difference in 6-month RNFL in the affected eye was 7.40 µm (per-protocol population), corresponding to 30% reduction in extent of RNFL loss in phenytoin compared with placebo
VISIONARY- MS (NCT03536559)	Phase 2, randomised, placebo-controlled, double-blind trial of nanocrystalline gold (CNM-Au8) in 150 people with MS and evidence of chronic optic neuropathy	Change in 2.5% LCLA; change in best-corrected LCLA (BC-LCLA)	Functional composite responder analysis; ff-VEP P100 latency and amplitude change; mf-VEP latency and amplitude change; change in RNFL, GCIPL thickness; NEI-VFQ-25	Terminated due to recruitment challenges
ReCOVER (NCT02521311)	Phase 2, randomised, placebo-controlled, double-blind trial of clemastine in 90 people diagnosed with acute demyelinating optic neuritis	Change in ff-VEP P100 latency; change in 2.5% Sloan LCLA	Change in RNFL thickness	Recruiting
ReWRAP (NCT04002934)	Phase 2, randomised, placebo-controlled, double-blind trial of bazedoxifene acetate (BZA) in 50 female patients with RRMS and prolonged baseline VEP latency	Change in ff-VEP P100 latency	NA	Recruiting
ONSTIM (NCT04042363)	Randomised, double-blind, sham-controlled trial assessing transorbital electrical stimulation in 45 people with RRMS and acute optic neuritis	Change in ff-VEP P100 latency	Change in ff-VEP amplitude; change in macular volume, RNFL and GCIPL thickness; change in mean deflection of visual field change	Recruiting
CCMR Two (NCT05131828)	Phase 2, randomised, placebo-controlled, double-blind trial of the combination of metformin and clemastine in 70 people with RRMS with chronic stable optic neuropathy	Change in ff-VEP P100 latency	Change in mf-VEP latency; change in mean lesional MTR; change in RNFL, RGCL/IPL thickness; change in visual acuity with Sloan 100%, 2.5% and 1.25%; change in saccadic latency parameters	Recruiting
RESTORE (NCT05338450)	Phase 2, randomised, placebo-controlled, double-blind trial of clemastine fumarate in 80 patients with RRMS and INO	Change in VDI and VDI-AUC	Other VDI index measures and infrared oculography parameters; change in HCVA and LCVA	Recruiting

DTI, diffusion tensor imaging; ff-VEP, full-field pattern-reversal visual evoked potential; GCIPL, ganglion cell-inner plexiform layer; HCVA, high-contrast visual acuity; INO, internuclear ophthalmoplegia; LCVA, low-contrast visual acuity; mf-VEP, multifocal VEP; MS, multiple sclerosis; MTR, magnetisation transfer ratio; NEI-VFQ-25, 25-item version of the National Eye Institute Visual Function Questionnaire; ON, optic neuritis; RGCL/IPL, retinal ganglion cell layer/inner plexiform layer; RNFL, retinal nerve fibre layer; VDI, versional dysconjugacy indices; VDI-AUC, VDI-area under the curve.

In the context of phase 2 remyelination clinical trials, we believe the ff-VEP remains the current standard outcome measure; it is a highly valid measure, having been confirmed to directly reflect myelin status in chronically demyelinated optic nerves,^43^ and appears sensitive given it has shown significant effects in three previous clinical trials.^18 19 21^ There is also a compelling rationale to include mf-VEP in remyelination trial design: it affords a more precise evaluation of the visual field, is unaffected by the potential confounds of macular over-representation and phase cancellation and was more sensitive than ff-VEP in a substudy of a remyelination trial.^25^ Other visual outcomes that might be impacted by remyelination (or represent its downstream consequences) have included the structural and functional measures of OCT and LCLA. Yet, while there is good evidence for monitoring change in best-corrected 2.5% LCLA, recent experience of OCT in remyelination and neuroprotection studies suggests that this is best deployed as a measure of visual health at study inclusion, rather than being used to measure change over short-duration trials. Recent evidence has also supported the inclusion of oculography to measure remyelination of single white matter tracts (such as the MLF) and of wider distributed neuronal networks.

There are, however, limitations to using visual measures as a sole readout for a clinical trial. First, to capture remyelination of the visual pathway, measurable demyelination is required at study entry. Consequently, there is a burden of screening to select only those participants with sufficient MS-related damage to measure treatment effects; for instance, in recruiting ReBUILD’s cohort of 50 participants, a further 75 were excluded as their screening VEP did not meet the study criteria. Second, there is an additional constraint to participant selection, as AON introduces a potential confound to visual measures. Therefore, it has become important to focus either on those following AON or on those with chronic optic neuropathy. Third, sample size calculations are confounded by uncertainty in what constitutes a clinically important effect. The objective of a remyelinating treatment is primarily to protect axons and so delay or prevent disability accumulation, which will likely only manifest over a period of many years. Identifying the magnitude of treatment effect on VEP latency, for instance, that translates into a clinically meaningful change to disability remains to be done. Finally, it should be acknowledged that not all lesions are equal in their capacity for remyelination, even within the same individuals.^107 108^ This within-patient heterogeneity is likely underpinned by regional differences in OPCs^109 110^ and the lesion environment.^111 112^ We should therefore be mindful that the degree of remyelination detected in the visual pathway may not translate directly to lesions elsewhere in the CNS. And it is possible that lesions in the visual pathway might be more or less responsive to remyelination-promoting drugs. On this account, myelin-sensitive MRI sequences provide more widespread information on tissue-specific damage and repair.

Our approach in remyelination trial design is therefore to include visual outcome measures, alongside those MRI measures that we believe will be most sensitive to remyelination: in CCMR One, perhaps the most compelling evidence of a biological effect of bexarotene was the alignment between the imaging and electrophysiological results.^18^ Given that sufficient axons are required for remyelination, we also direct our studies to those more likely to demonstrate an effect, for instance, by recruiting those with RRMS, while excluding eyes with significant axon loss on OCT. We also believe that remyelination will be greatest in younger individuals^113^ and so strongly support the use of rejuvenating drugs^114^ and analyses that account for the impact of age.
